# Influence of Magnetostriction Induced by the Periodic Permanent Magnet Electromagnetic Acoustic Transducer (PPM EMAT) on Steel

**DOI:** 10.3390/s21227700

**Published:** 2021-11-19

**Authors:** Cong Zhu Sun, Anthony Sinclair, Tobin Filleter

**Affiliations:** Department of Mechanical and Industrial Engineering, University of Toronto, 5 King’s College Road, Toronto, ON M5S 3G8, Canada; sinclair@mie.utoronto.ca (A.S.); filleter@mie.utoronto.ca (T.F.)

**Keywords:** electromagnetic acoustic transducer, periodic permanent magnet, magnetostriction, Lorentz, shear horizontal wave, steel

## Abstract

The periodic permanent magnet electromagnetic acoustic transducer (PPM EMAT) is a sensor that can generate and receive shear horizontal (SH) waves without direct contact with the inspected medium using the Lorentz mechanism. However, the PPM EMAT experiences high signal variance on ferromagnetic steel under specific conditions, such as a change in signal amplitude when the sensor is moved in the direction of SH wave propagation. Magnetostriction effects are hypothesized to be the cause of these anomalous behaviors; the objective of this paper is to determine the relative strengths of the magnetostriction and Lorentz wave generation mechanisms for this type of EMAT on steel. This goal is accomplished through the use of a second EMAT, which induces only magnetostriction (MS-EMAT), to calibrate a novel semi-empirical magnetostriction model. It is found that magnetostriction effects reduce the amplitude of the SH wave generated by this particular PPM EMAT transmitter by an average of 29% over a range of input currents. It is also determined that magnetostriction is significant only in the investigated PPM EMAT transmitter, not the receiver. In terms of practical application, it is shown that the MS-EMAT is less sensitive to changes in the static and dynamic fields than PPM EMATs at specific operating points; this makes the MS-EMAT a viable alternative for nondestructive evaluation despite lower amplitudes.

## 1. Introduction

Aboveground storage tanks and pipes are large steel structures that experience corrosion and can be inspected with guided waves using electromagnetic acoustic transducers (EMATs) [1]. EMATs are rapid and economical sensor solutions for detecting significant thickness reductions due to corrosion, as they consume little power and do not require direct contact with the inspected medium. For inspecting plate structures, using a periodic permanent magnet (PPM) EMAT has been a popular choice [2,3,4,5,6] due to their high signal-to-noise ratio and ability to generate shear horizontal (SH) ultrasonic waves. SH waves are useful for nondestructive testing (NDT) since all particle displacements are parallel to the plane, reducing the influence of surface coatings and fluid contact. Andruschak et al., used PPM EMATs to generate SH waves near the inflection point of the dispersion curve to optimize the detection of defects in the presence of support contacts [2]. Hirao and Ogi have developed an EMAT technique utilizing the PPM EMAT for detecting corrosion defects on steel pipelines by measuring the amplitude and phase of the detected SH wave signals [3]. Trushkevych et al., used miniaturized PPM EMATs with a robot to detect wall thinning on steel [4]. Shi et al., proposed a method for detecting circumferential cracks on a pipe with SH waves produced by a PPM EMAT by analyzing the reflections from the edges of the defect [6].

An alternate method of generating SH waves is the magnetostrictive (MS) EMAT, if the inspected medium is ferromagnetic. The design was first proposed by Thompson [7]. However, these EMATs are utilized less frequently due to their lower efficiency [8] and reliance on a nonlinear phenomenon instead of the linear mechanism used by the PPM EMAT. Nonetheless, there have been investigations into SH wave MS-EMATs. Zhang et al., have formulated an improved analytical model of the MS-EMAT with higher prediction accuracy of multiple parameters [9]. Wei et al., made an omnidirectional MS-EMAT for SH wave guided wave tomography to detect defects in steel plates [10]. An alternate design based on a ferrite core has been developed for both Lamb and SH wave generation by Kwun and Kim [11].

This project uses the lowest mode (SH0) because it is non-dispersive: an operator looks for significant amplitude changes to the received pulse in a pitch-catch configuration. Although only plate waves are investigated here, these results are also applicable to pipes inspected in the circumferential direction as a first-order approximation [12].

For the PPM EMAT to apply to NDT, it should produce the same stable signal while stationary or moving across the structure’s surface in any direction. An inspection system consisting of a PPM EMAT transmitter to PPM EMAT receiver is tested on a mild steel plate during preliminary testing. It is found that the received signal fluctuates as the inspection system is moved along the surface of the plate in the direction of wave propagation [13]. The PPM EMAT is based on the Lorentz mechanism, which is not affected by steady movement along the wavelength direction. This observation is verified using the same system on an aluminum plate, where the same inspection system does not exhibit any movement effects.

It is suspected that magnetostriction, a mechanism applicable to ferromagnetic steels, might be operating alongside the Lorentz mechanism. By comparing magnetostriction and Lorentz-based EMATs, Ribichini et al., found that PPM EMAT transmitters generally produce a larger amplitude per unit current than magnetostriction-based EMATs [8]. Ashigwuike et al., found that at high amplitudes of static field and input current amplitude, the dynamic Lorentz force outperforms other transduction mechanisms in either EMAT configuration. However, the Ribichini tests were performed on a 0.5 mm steel plate, which is relatively easy to saturate with permanent magnets and is much thinner than the plate used in many critical structures, such as aboveground storage tanks or pipes. (Most large aboveground storage tanks have a minimum nominal plate thickness of 1/4″(6.35 mm) [14] (p. 161)). The Ashigwuike simulations showed that the dynamic and static Lorentz forces produce significantly higher particle displacements than the magnetostrictive strains when the input current is in the hundreds of amperes. In a typical NDT application, the pulser/receiver unit must be light and portable; this significantly limits the maximum input current to the transmitter coil, such that it is not possible to saturate the plate with a magnet array of appropriate physical size. Additionally, Thompson [5] stated that magnetostriction is significant relative to Lorentz at lower bias field strengths. Therefore, there is no conclusive evidence that Lorentz effects are significantly stronger than magnetostriction mechanisms in a portable PPM EMAT transmitter; this is despite the fact that the published literature suggests that magnetostriction is insignificant in general PPM EMATs [8,9,10,11,12,13,14,15].

On the contrary, Karimi found that magnetostriction could play a significant role in a PPM EMAT transmitter [13] on steel; she found that the amplitude of the magnetostrictive contribution to the total transmitted signal was approximately 55% of the Lorentz contribution in the static case. Her measurements also showed that the signal amplitude varied by +/−20% when the entire transmitter–receiver assembly was in motion parallel to the direction of wave propagation. The behavior could only be modeled with a combination of magnetostriction and Lorentz mechanisms. It was shown that magnetic hysteresis causes the PPM-induced bias field within the steel plate to undergo large magnetization cycles and become distorted in shape. Since the bias field is shifted, there is consequently an induced spatial shift in the magnetostriction induced regions with respect to the Lorentz induced regions, causing the movement variations [13]. However, her modeling work was not complemented by sufficient experimental results to substantiate her results for the static case.

This paper aims primarily to determine the relative magnitudes of Lorentz and magnetostriction contributions in a particular PPM EMAT transmitter operating on mild steel plates. Other PPM EMATs may have similar magnetostriction to Lorentz trends but not necessarily the exact results. These two wave generation mechanisms are separated through a combination of both PPM and magnetostrictive EMATs to generate SH0 waves and lead to a semi-empirical numerical model for including a magnetostriction effect in PPM EMAT transmitters. Three dimensional Finite Element (FE) analysis is used in addition to experimental data to determine parameter values for the model. It is found that magnetostriction decreases the wave amplitude generated by the PPM EMAT transmitter by an average of 29%.

A secondary objective is to compare the particular PPM EMAT in the study with the MS-EMAT transmitter in signal stability. The model and experimental data are used to explore the relative merits of PPM EMATs and MS-EMATs to generate ultrasonic SH waves for NDT. It is found that the MS-EMAT is less sensitive to input variations than the PPM EMAT.

## 2. Materials and Methods

### 2.1. Ferromagnetic Properties of 1018 Steel

A36 steel is commonly used on aboveground storage tanks and pipes, but accurate values of its magnetic properties are not readily available. Therefore, values for AISI 1018 steel are often used instead. The chemical compositions and mechanical properties are similar between A36 and 1018, such that several EMAT researchers have used magnetic properties of 1018 steel [16,17,18].

A solution of the electromagnetic and magnetostrictive equations on ferromagnetic materials requires data pertaining to both the static case (conventional B-H curve) and dynamic case (magnetic permeability), as well as magnetostriction coefficients. The steel plate used here for experimental measurements is assumed to follow the SAE1018 B-H static curve and material properties defined within the COMSOL finite element program [19], allowing for the calculation of static magnetic fields within the plate and wave propagation in a FE simulation.

Magnetostriction is the strain induced by an applied magnetic field. The magnetostrictive response of 1018 steel to an applied field was measured by Thompson [17]. The curve was found under static conditions.

Under high-frequency oscillating fields, the magnetic permeability must be specified, as the B-H curve does not describe the steel’s behavior under these conditions. There are data on the transverse isotropic permeability, where the static field is much larger than and perpendicular to the dynamic field. The transverse isotropic permeability is found from empirical data from [18], which considers frequency and static field strength. The EMATs in this study are operated at 0.25 MHz, but permeability data corresponding to that frequency is not available. Therefore, the closest published data is used instead, corresponding to a 0.35 MHz small dynamic field superimposed on a 0.4–1 kA/m static field. Despite the dynamic magnetic fields being significantly larger than the static field in the current EMATs, the assumption is made that the 0.35 MHz permeability curve is applicable.

### 2.2. SH Wave EMATs

There are two different SH wave generation devices for steel used in this project: PPM EMAT and MS-EMAT. Although the PPM EMAT is the primary target of the investigation, the MS-EMAT is needed to isolate and quantify the magnetostriction wave generation mechanism.

#### 2.2.1. PPM EMAT

The Lorentz model applied to the PPM EMAT is well-known. Maxfield et al. [20] analyzed a Lorentz force EMAT made with rare-earth cobalt magnets. Thompson et al. [21] modeled a Lorentz force EMAT operating on a ferromagnetic test plate. The static field Lorentz force $F_{L}$ is given by

(1) $$F_{L}=J\times B$$

where J is the current density and B is the static magnetic flux density vector. The dynamic field Lorentz force is not included as it operates at double the frequency of the input current to the transmitter coil. In the steel specimen directly below the receiver EMAT, the current density is

(2) $$J_{L}=\sigma_{E}v\times B$$

where v is the velocity vector of the particles within the steel plate.

**Figure 1:**
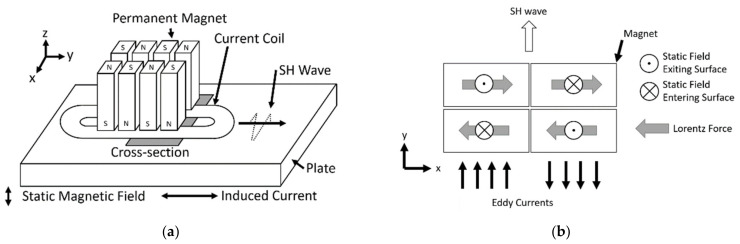
(**a**) Periodic Permanent Magnet (PPM) EMAT 3D Diagram; (**b**) Cross-section of PPM EMAT at Surface.

A schematic of the PPM EMAT is shown in Figure 1a. Using a magnet array and a racetrack coil, the PPM EMAT generates an alternating pattern of Lorentz forces (Figure 1b) in the steel directly below the transmitter. This pattern then produces SH waves.

In highly conductive materials, if the skin depth is significantly smaller than the ultrasonic wavelength, the Lorentz force is less sensitive to the magnetic permeability and conductivity of the plate than to the magnetostriction [5].

#### 2.2.2. MS-EMAT

**Figure 2:**
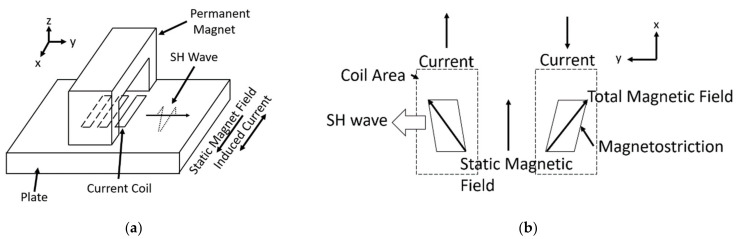
(**a**) Magnetostrictive (MS) EMAT 3D Diagram; (**b**) Top View of the Magnetostriction within the Plate.

An alternate option for generating SH waves in ferromagnetic materials is the MS-EMAT. It is used in this paper to calibrate the magnetostriction model within the operating regime of the PPM EMAT configuration. Figure 2a shows the components of the MS-EMAT, which are primarily a horseshoe magnet and a meander-shaped coil. The combination of static and dynamic magnetic fields induced by the magnet and alternating current in the transmitter coil, respectively, induces magnetostrictive strains in a periodic pattern in the steel specimen. These then produce SH waves (Figure 2b). Unlike the Lorentz mechanism, magnetostriction has a highly nonlinear response to the input static and dynamic magnetic fields; experiments by Murayama showed that different static field values are optimal for the transmitter and receiver [22].

Magnetostriction is a nonlinear phenomenon but often linearized with an analogy to piezoelectricity [9,15,23,24]. The linearized magnetostriction model [25] (p. 48) consists of two equations

(3) S=Cσ+dH

(4) $$B=d^{T}\sigma +\mu^{\sigma }H$$

where S is the strain tensor (6 × 1 in Voigt notation), C is the compliance matrix (6 × 6), σ is the stress tensor (6 × 1), d is the magnetostrictive coefficient matrix (6 × 3), and H is the magnetic field strength vector (3 × 1). Only the dynamic components are relevant for wave generation/reception, such that static forces can be neglected. The material is assumed to be isotropic, and magnetostriction is assumed to be isovolumic. The model has been experimentally validated for EMAT performance on nickel plate [26], which is another ferromagnetic material. However, there is no existing magnetostriction model for steel at low static fields relative to dynamic fields, typical of most PPM EMATs on steel.

### 2.3. Magnetostriction within the PPM EMAT

Since the coil in both the MS-EMAT and PPM EMAT configurations runs parallel to the plate surface, the main difference is the orientation of the coil lines with respect to the static field inside the plate. For a Lorentz-based EMAT, the static field in the plate must be perpendicular to the direction of the current in the coil, while in the MS-EMAT, the current runs parallel to the static field.

**Figure 3:**
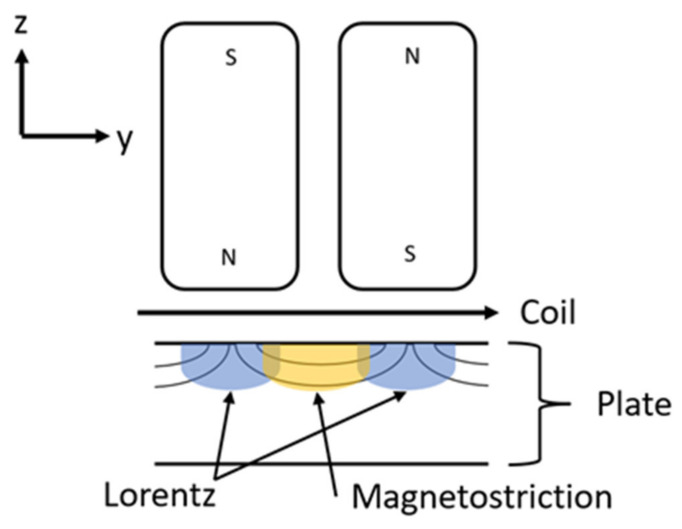
Lorentz and Magnetostriction mechanisms within the PPM EMAT (Side View).

Both Lorentz and magnetostriction mechanisms occur within a PPM EMAT that operates on ¼″ (6.35 mm) thick steel plates. While the static field is perpendicular to the surface of the plate when it enters the plate, the shortest magnetic path in a permeable material towards the opposite pole is along the surface of the plate (Figure 3). Therefore, a significant portion of the static field is oriented parallel to the main lines of the racetrack coil; that geometry induces magnetostriction in the steel, such that both magnetostriction and Lorentz effects lead to the generation of SH waves.

It is difficult to use only experimental methods to determine the significance of each mechanism within the PPM EMAT, as both mechanisms are excited by the same coil and magnetic field pattern. As a result, each mechanism simultaneously produces SH waves at the same frequency and wavelength. Measuring high-frequency strains within the plate underneath the PPM EMAT, is also a challenge. With direct measurements being impractical, a numerical magnetostriction model that applies to both the PPM EMAT and MS-EMAT should be derived instead. Next, the magnetostriction model for the transmitter can be calibrated using the MS-EMAT (pure magnetostriction mechanism) experimental data in addition to PPM EMAT (both magnetostriction and Lorentz mechanisms) readings. Finally, the individual components of the total PPM EMAT numerical simulation can be isolated to determine the relative strengths of the magnetostriction and Lorentz mechanisms.

### 2.4. PPM EMAT Magnetostriction Model

A valid magnetostriction model should be applicable to various EMAT configurations, including both PPM and MS-EMATs. To that end, the conventional linear magnetostriction model [23] (p. 26) is extended to include two static field components. In addition, the high static bias field assumption of the conventional linear model needs to be excluded as it does not apply to all EMAT configurations.

**Figure 4:**
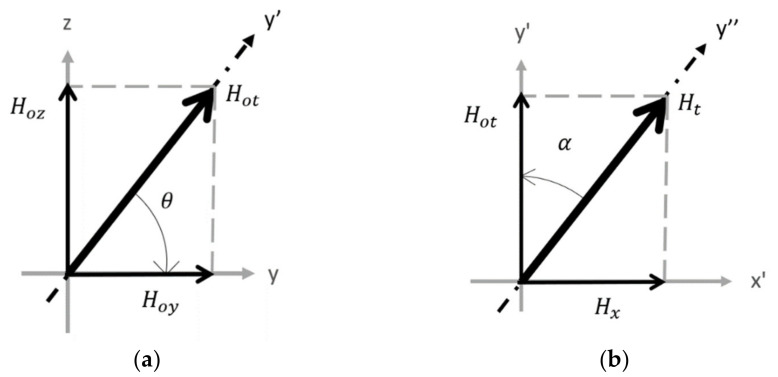
(**a**) Component Breakdown of the Static Field; (**b**) Dynamic Field Angle α Definition.

**Figure 5:**
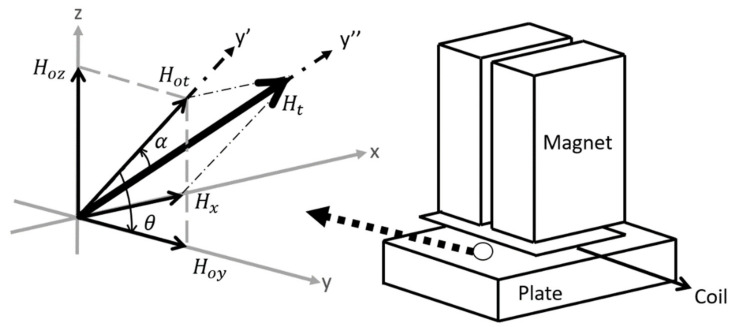
3D Diagram of the coordinate systems with a section of the PPM EMAT.

From Figure 3, the PPM array produces two major static field components: the y-component ($H_{oy}$) and the z-component ($H_{oz}$). (The x-component is insignificant in terms of generation of SH waves and neglected from the calculation.) There are three coordinate systems: x-y-z, x’-y’-z’, and x”-y”-z”. The basis vectors of the x”-y”-z” system are in the three principle strain directions, while the x’-y’-z’ system is an intermediate system between the x-y-z and x”-y”-z” systems. The total static field vector is represented by its magnitude $H_{ot}$ and directional angle θ in the y-z plane (Figure 4a); the total field (static field plus dynamic field) can be represented by its magnitude $H_{t}$ and directional angle α (Figure 4b). A 3D diagram of both coordinate systems along with one side of a PPM EMAT is depicted in Figure 5. The angles can be defined as

(5) $$\alpha =\tan^{-1}\left(\frac{H_{x}}{H_{ot}}\right)$$

(6) $$\theta =\cos^{-1}\left(\frac{H_{oy}}{H_{ot}}\right)$$

Some assumptions regarding our magnetostriction model are retained from the original linear model of reference [23]. Magnetostrictive strain is assumed to be oriented along the direction of the total field vector, and magnetostriction is assumed to be an equivoluminal process. Polycrystalline materials, such as steel, can be considered to be isotropic [27] p. 355, because the average magnetic domain size is much smaller than the ultrasonic wavelength.

The magnetostrictive strain **S″** in the principal coordinate system (x”-y”-z”) is [23] (p. 27)

(7) $$S^{\prime \prime }=\left[\begin{array}{ccc} -\frac{1}{2}\epsilon_{t} & 0 & 0\\ 0 & -\frac{1}{2}\epsilon_{t} & 0\\ 0 & 0 & \epsilon_{t} \end{array}\right]$$

where $\epsilon_{t}$ is the total magnetostriction in the *z*” direction. The magnetostrictive strain tensor is transformed to the global coordinate system (x-y-z) from its principal coordinate system by two consecutive rotations so that it can be related to both the static and dynamic electromagnetic fields exerted by an EMAT’s magnets and coils

(8) $$S=R_{x}R_{z}S^{\prime \prime }R_{z}^{T}R_{x}^{T}$$

where

(9) $$R_{z}=\left[\begin{array}{ccc} \cos\alpha & \sin\alpha & 0\\ -\sin\alpha & \cos\alpha & 0\\ 0 & 0 & 1 \end{array}\right]$$

(10) $$R_{x}=\left[\begin{array}{ccc} 1 & 0 & 0\\ 0 & \cos\theta & -\sin\theta \\ 0 & \sin\theta & \cos\theta \end{array}\right]$$

Rotating the strain tensor is easier in its 3 × 3 form but will be referred to its Voigt notation form (6 × 1) for the rest of the derivation. Next, the strain tensor is differentiated with respect to $H_{x}$, $H_{y}$, and $H_{z}$ to find the magnetostrictive coefficients

(11) $$d_{Ij}=\frac{\delta S_{I}}{\delta H_{j}}$$

where I=1, 2, 3…6 and j=x,y,z. This study is focused on SH waves (xy-component) and the PPM EMAT’s racetrack coil produces a magnetic field oriented primarily in the x-direction. Therefore, only the xy-component (component 6 in Voigt notation) induced by an x-direction field, $d_{6x}$, is needed for this investigation

(12) $$d_{6x}=\frac{3}{2}\;\gamma \sin\alpha \sin2\alpha \cos\theta +\frac{3\epsilon_{t}}{H_{ot}}\cos2\alpha \cos^{2}\alpha \cos\theta$$

where γ is the slope of the magnetostriction curve at the total magnetic field strength point. Magnetostriction is hysteretic [28], so the strain is not precisely aligned with the applied field. It is observed from preliminary experiments that the theoretical model does not agree well with experimental MS-EMAT data, which means that nonlinear effects are significantly affecting the non-hysteretic model. The average impact of nonlinear effects such as hysteresis can be approximated through the use of empirical factors added to estimated values of $d_{6x}$. This yields a semi-empirical equation for the key magnetostrictive coefficient relevant to the PPM EMAT

(13) $$d_{6x}^{PPM}=\frac{3}{2}\;\gamma_{M}\sin\alpha_{M}\sin2\alpha_{M}\cos\theta +\frac{3\epsilon_{t}f_{\epsilon }}{H_{otM}}\cos2\alpha_{M}\cos^{2}\alpha_{M}\cos\theta$$

where

(14) $$H_{otM}=H_{ot}\left(f_{Hot}\left(H_{ot}-H_{ot_{norm}}\right)+1\right)$$

(15) $$\alpha_{M}=f_{\alpha }\tan^{-1}\left(\frac{\left|H_{x}\right|}{H_{otM}}\right)$$

(16) $$\gamma_{M}=f_{\gamma }\frac{\epsilon_{t}-\epsilon \left(H_{s}\right)}{H_{t}-H_{otM}}$$

Equations (13)–(16) can also be applied to a model of the MS-EMAT with its respective coefficient ($d_{6y}^{MS}$) by setting θ=0 in Equation (13), with only a slight change to Equation (15)

(17) $$\alpha_{M}^{MS}=f_{\alpha }\tan^{-1}\left(\frac{\left|H_{y}\right|}{H_{otM}}\right)$$

$H_{s}$ is the point of peak static magnetostriction on the magnetostriction curve, which is approximately 5000 A/m on the 1018 steel magnetostriction curve [17]. $H_{ot_{norm}}$ is the center point for Equation (14), which empirically modifies the value of $H_{ot}$ to $H_{otM}$ to better account for the change in signal amplitude that is caused by the static field. $H_{ot_{norm}}$ is set to 797 A/m for the PPM EMAT and MS-EMAT configuration in this study to simplify the finding of the empirical factors. $f_{\gamma }$, $f_{\alpha }$, $f_{\epsilon }$, and $f_{Hot}$ are empirical fitting factors to account for hysteresis effects. $f_{\gamma }$ modifies the magnitude of the magnetostriction curve calculation in Equation (16). $f_{\alpha }$ decreases the angle between the static and dynamic field (Equation (15)) caused by the lag between the strain and applied field. $f_{\epsilon }$ modifies the magnitude of the magnetostriction data from the static magnetostriction curve ($\epsilon_{t}$). $f_{Hot}$ adjusts the value of the static magnetic field strength used to calculate the magnetostriction coefficient in Equation (14). The empirical factors are found by fitting the model predictions with experimental data from MS-EMATs and PPM EMATs. $\alpha_{M}$ and $\gamma_{M}$ are the α and γ values modified by $f_{\alpha }$ and $f_{\gamma }$, respectively.

The proposed model’s restrictions are:

The total field must be greater than the inflection point of the specimen’s magnetostrictive curve ($H_{s}$). This limitation is due to the formulation of Equation (16), which does not apply when $H_{t}<H_{s}$. Most commercial EMATs are pulsed at high currents for high signal levels, which usually satisfies this condition.

The model only predicts the xy-component of the shear and stress driven by a dynamic field in a single dominant direction. These are satisfied by SH wave EMATs.

The model’s results can only be considered valid for the static and dynamic fields produced by the EMATs in the experimental data used to derive the empirical factors. A new set of empirical factors to account for hysteresis and other nonlinear phenomena would need to be found for other operating regimes. Equation (13) can only be applied in a limited area underneath the EMAT footprint. If applied across the entire geometric space beyond that limited area, results are poor where there are very low dynamic or static fields.

#### Magnetostriction Model Implementation Procedure

**Figure 6:**
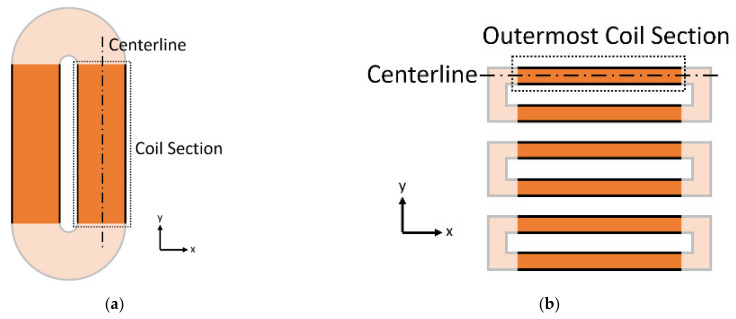
Top View Coil Section Diagram for (**a**) PPM EMAT; (**b**) MS-EMAT.

The first step to employ the new model is to solve the static magnetic field using a finite element program such as COMSOL Multiphysics. In the PPM’s case, the $H_{ot}$ values at the surface of the plate (centerline underneath each row of magnets, as seen in Figure 6a) are used for the entire sensor as a y-dependent value. A simplification can be made for the MS-EMAT’s case: As $H_{ot}$ produced by the horseshoe magnet is nearly constant beneath the coil, $H_{ot}$ is averaged over the entire transmitter coil area, within the plate volume.

Next, another set of frequency-domain magnetic field simulations are performed at the current burst’s center frequency. The dynamic field originating from the alternating current in the transmitter coil is solved; the input current to the coils inside the simulation is the average amplitude of the experimental current in the time domain. $\alpha_{M}$ can then be found. The range of $\alpha_{M}$ values must be reduced to a single effective $\alpha_{M}$ value for each EMAT experimental case to allow for simple derivation of magnetostriction empirical factors later.

For the PPM EMAT, a single average value for $\alpha_{M}$ is determined from Equation (15) under the centerline of the racetrack coil (Figure 6a), as that location contains the highest dynamic fields and, thus, accounts for a majority of the wave generation. For the MS-EMAT, Equation (17) is used to find a single effective value of $\alpha_{M}$, determined using the centerline of the outermost section of the meander coil. (Simulation tests indicate that it is the outermost sections (Figure 6b) of the meander coil that contribute the most to the SH wave generation).

Determining a value for $H_{t}$ to be used in finding values for $\epsilon_{t}$ and $\gamma_{M}$ (Equation (16)) follows the same guiding principles as for the determination of $\alpha_{M}$: An effective average value for $H_{t}$ is computed under the centerline coil section (Figure 6a) for the PPM EMAT or centerline outermost coil section (Figure 6b) for the MS-EMAT, on the surface of the plate.

As a consequence of these averaging operations, all regions under the transmitter coil for the MS-EMAT should share the same $d_{6y}^{MS}$ value. For the PPM EMAT, the value for $d_{6x}^{PPM}$ varies along the y-direction only.

### 2.5. Finite Element Modeling of Entire NDT System to Determine Magnetostriction Empirical Factors

The entire EMAT transmitter, propagation of SH waves inside the plate, and receiver are simulated in the frequency domain using COMSOL Multiphysics 5.6. The transmitter, wave propagation, and receiver are simulated separately to reduce memory requirements. Feedback effects are assumed to be insignificant due to the low efficiency of EMATs and distance between the EMATs (300 mm). The 1018 steel test plate is sized to be much larger than the EMATs (914.4 mm × 304.8 mm × 6.35 mm thick) so that the reflected waves do not interfere with the results. There is a transmitter and receiver, spaced 300 mm apart. This approach has been used in the literature to model EMAT performance [16,26,29,30,31,32]. Two sets of simulations with the MS-EMAT and PPM EMAT transmitters are performed, but only the PPM EMAT receiver receives both sets.

The modeling procedure is similar for both the MS-EMAT and PPM EMAT transmitter. First, a static magnetic field simulation is run without the influence of the coil to find the magnetic flux density or field strength. These static fields are needed as input to both the Lorentz and magnetostriction equations for the PPM EMAT, or just the magnetostriction equation for the MS-EMAT. Next, a dynamic frequency-domain magnetic field simulation is run; this only includes the active meander (MS-EMAT) or racetrack (PPM EMAT) coils and plate. The input to the coil for the simulation is the Fourier transform of the experimentally measured input current. All input current waveforms have a bandwidth of 100 kHz, which is discretized into 5 kHz bins. No major changes to the receiver voltage waveform are observed when the simulations are discretized at 1 kHz instead of 5 kHz.

The Lorentz and magnetostriction mechanisms within the PPM EMAT transmitter are solved separately, and the total output is summated to yield the transmitted wave. With the Lorentz mechanism, the induced forces can be calculated directly from separate frequency-domain and static simulation results, then used as input into an elastic simulation. For magnetostriction, the electromagnetic and elastic equations are coupled and are solved together. The MS-EMAT has no significant Lorentz components due to the geometry of the horseshoe magnet and meander coil, so only the magnetostriction is solved with FE.

**Figure 7:**
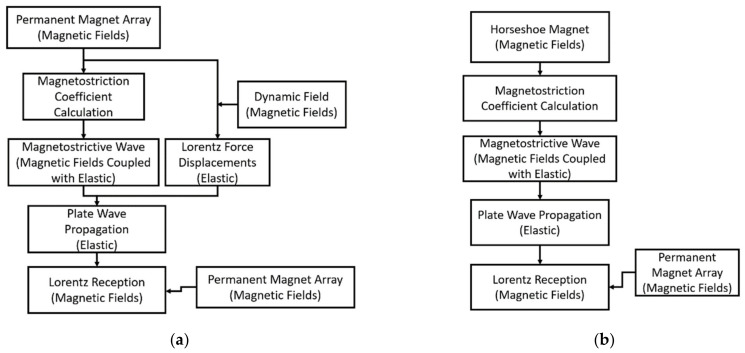
Block Diagrams of the Simulation Process (**a**) PPM-to-PPM; (**b**) MS-to-PPM.

After the transmitter simulation is complete, the displacements are inserted into a wave propagation simulation containing only the plate to find the displacements at the PPM EMAT receiver. Finally, those displacements are used for the PPM EMAT receiver simulation to find the receiver voltage. The block diagrams of the simulation processes are shown in Figure 7.

The MS-EMAT’s horseshoe magnet is meshed using tetrahedral elements. Symmetry is used to reduce the problem to quarter size with suitable boundary conditions. The magnet assembly and plate are meshed at 2 mm, but element size is reduced to 0.3 mm where the field exits the magnet and enters the plate. The elements away from the critical areas have a growth rate of 1.5 up to a maximum element size of 61 mm.

The electromagnetic domain within the plate is limited to three times the skin depth, which is substantially thinner than the plate (6.35 mm). Regions deeper than three times the skin depth and areas away from either transmitter or receiver coils contain negligible eddy currents and can be solved with only elastic equations. At least three elements per skin depth are required to accurately represent the dynamic magnetic field [25], so ten elements through the thickness of the plate are used in the frequency-domain simulation meshes; these elements are distributed so that elements are small and close to the surface to model the dynamic field decay with depth accurately.

The dynamic MS-EMAT mesh has eight elements per wavelength as there are no substantial improvements in ultrasonic wave simulation from a finer element size [33]. The meshes are comprised of hexahedral elements, which are more memory efficient than tetrahedral elements for rectangular shapes. A ring of perfectly matched layers (PMLs) absorbs acoustic waves that impinge the boundaries of the modeled region, such that the wave propagation simulation approximates that of an infinite plate [19]. The PML ring needs at least seven elements per SH wavelength to suppress any ultrasonic wave reflections [34]. The total PML here is one wavelength deep and contains ten element layers.

The dynamic PPM EMAT mesh is sized in the same way as the dynamic MS-EMAT mesh, with eight elements per wavelength and a PML ring of ten elements. Since the dynamic field requires a finer mesh than the static field, the dynamic mesh can be reused for the static field simulation without a loss in accuracy. The transmitter and receiver PPM EMAT mesh are identical.

The wave propagation mesh includes the footprint of the MS-EMAT or PPM EMAT transmitter along with the PPM EMAT receiver footprint, spaced 300 mm apart. In all cases, the mesh is identical because the footprints are identical. The plate is surrounded by a PML ring with ten elements to suppress reflections from the edges.

### 2.6. Experimental Setup

A PPM EMAT system is made for inspecting a 914.4 mm × 304.8 mm × 6.35 mm thick 1018 steel test plate. There is a transmitter and receiver, spaced 300 mm apart. The transmitter is designed to induce SH0 waves, with a center frequency of 250 kHz at 13.1 mm wavelength, minimizing the space between magnets (an area that induces magnetostriction). The racetrack coil has 30 turns. The PPM receiver is identical to the transmitter.

An MS-EMAT transmitter with a 12-turn meander coil is designed to run at the same wavelength, frequency, and on the same section of steel as the PPM EMAT transmitter to gather magnetostriction data while minimizing the number of variables. The same PPM receiver as used for the PPM transmitter is used for this system, such that the effects of only switching transmitters can be assessed.

**Figure 8:**
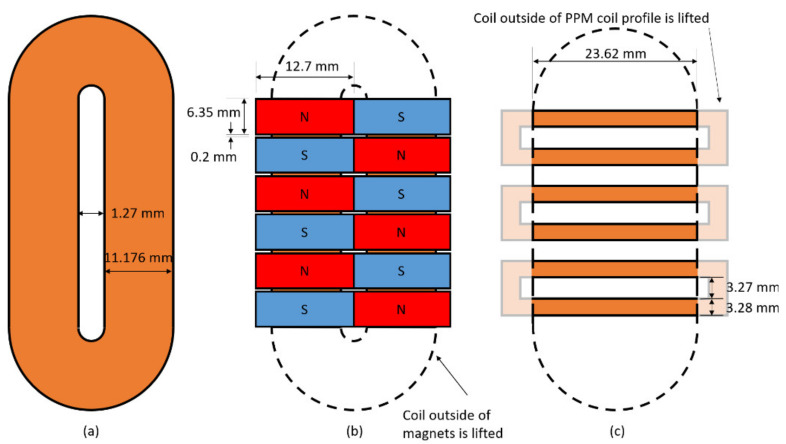
Overhead View and Dimensions of each EMAT Configuration. (**a**) Racetrack coil (PPM with Magnets Removed); (**b**) PPM EMAT; (**c**) Meander coil in the MS-EMAT transmitter. “Lifted” portions of coils are sufficiently distant from the test plate that they do not participate in the transduction mechanism.

**Figure 9:**
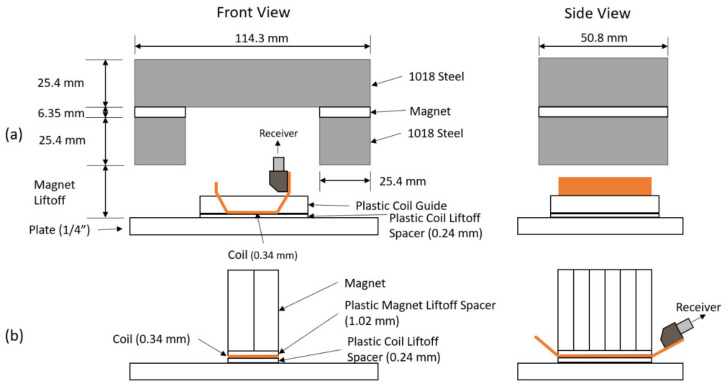
EMAT Front and Side Views: (**a**) MS-EMAT; (**b**) PPM EMAT.

Schematics of both EMAT transmitters are shown in Figure 8 and Figure 9. The coil segments outside of the magnet footprint are lifted to reduce the eddy currents they generate. In the case of the MS-EMAT, the liftoff of those exterior coil segments eliminates any Lorentz components.

The PPM itself is made of 12.7 mm × 6.35 mm × 25.4 mm thick N42 magnets. The horseshoe magnet for the MS-EMAT comprises two 50.8 mm × 25.4 mm × 6.35 mm thick N52 magnets and 1018 steel blocks. Both EMAT coils have 0.34 mm thick, with active sections at 0.24 mm liftoff.

A PowerBox H (PBH) system (Innerspec Technologies, Forest, VA, USA) is used to pulse the transmitter EMATs and amplify the receiver voltage. The input current is measured with a Tektronix P6022 current probe. The PBH is set to time-average the receiver voltage 16 times, with a pulse repetition frequency of 15 Hz. Custom impedance matching circuits are designed to maximize the received signal.

The MS-EMAT experiments are designed to cover the entire range of magnetic field values induced by this study’s PPM EMAT configuration so that the empirical factors for the new magnetostriction model can be determined. Since field measurements inside the plate are not possible, the static field strength on the surface of the plate produced by the PPM is simulated and found to range from 540–820 A/m under the centerline of each row of magnets.

Measurements are made at three values of magnet liftoff to cover this range of static fields with the MS-EMAT transmitter. The horseshoe magnet is much larger than the MS-EMAT coil, generating uniform fields, allowing the use of averaged values within the coil area. Values of the static magnetic field $H_{ot}$ are determined via COMSOL simulation and verified by a Gauss meter in air just above the test plate:

1. MS-EMAT magnet liftoff = 4.45 mm; associated average static magnetic field $H_{ot}$ = 1084 A/m.
2. MS-EMAT magnet liftoff = 8.92 mm; associated average static magnetic field $H_{ot}$ = 797 A/m.
3. MS-EMAT magnet liftoff = 23.68 mm; associated average static magnetic field $H_{ot}$ = 437 A/m.

The maximum static magnetic field variation under the MS-EMAT transmitter within the plate is ±6.5% of the average value for all three liftoff values within the MS-EMAT coil area profile and with depth into the plate.

The PPM EMAT’s input current is five cycles of a sine wave, with average peak currents of 4.8 A, 9.9 A, and 20.5 A. This waveform has a bandwidth of approximately 100 kHz. The maximum dynamic magnetic field produced by the racetrack coil within the plate is determined by COMSOL simulation to range from $8.6\times 10^{3}$ A/m (4.8 Amperes input current) up to $3.65\times 10^{4}$ A/m (20.5 Amperes input current). To cover a similar range of field values, the MS-EMAT’s average input current is set to range from 5.8 A ($7.2\times 10^{3}$ A/m) to 35.1 A ($4.37\times 10^{4}$ A/m) for each of the three values of static field $H_{ot}.$ Measurements and COMSOL simulations are then made for three EMAT system configurations:

- PPM EMAT transmitter to PPM EMAT receiver (PPM-to-PPM)
- MS-EMAT transmitter to PPM EMAT receiver (MS-to-PPM)
- one MS-EMAT transmitter to MS-EMAT receiver (MS-to-MS) experiment

**Figure 10:**
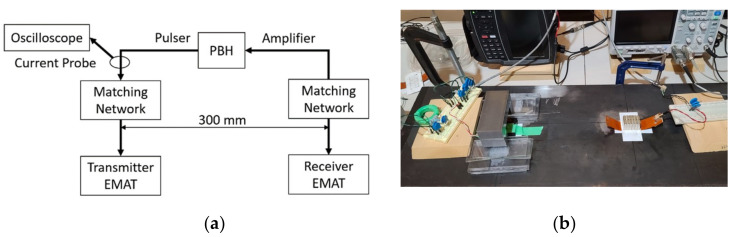
Typical Experimental Setup (MS-to-PPM shown) (**a**) Schematic; (**b**) Photograph.

Each EMAT configuration must be run on the exact same steel footprint to avoid the effects of non-homogeneities in the steel sample. Temporal signal averaging produced a very stable and repeatable output waveform. A typical experimental setup is shown in Figure 10.

## 3. Results

### 3.1. Magnetostriction in the Receiver

**Figure 11:**
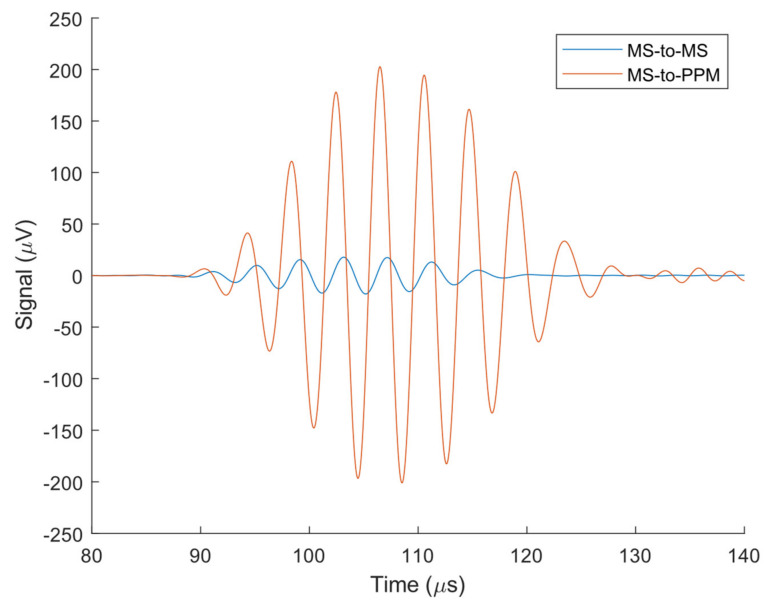
Experimental MS-to-MS and MS-to-PPM Receiver Voltage on Steel Plate. Both MS-EMAT transmitter and receiver have a magnet liftoff of 8.92 mm. The input current to the transmitter in both tests is the same waveform with a maximum of 35.1 A.

First, the magnetostriction in the PPM EMAT receiver is estimated to determine if it is significant enough to be modeled. An MS-to-MS experiment is compared against an MS-to-PPM experiment with the same MS-EMAT magnet liftoff (8.92 mm) for both transmitter and receiver and input current (35.1 A) in the transmitter (Figure 11).

The component of the static field that is tangential to the plate’s surface has a peak value close to the average tangential static field produced by the MS-EMAT’s horseshoe magnet. Thus, the MS-EMAT receiver induces a magnetostriction coefficient close to the peak magnetostriction coefficient that the PPM EMAT produces between the magnets. However, as the PPM EMAT produces a lower average tangential static field across the same footprint, it does not induce nearly as much magnetostriction as the MS-EMAT receiver. In other words, the total voltage induced through magnetostriction in the PPM EMAT receiver is less than the total voltage induced by the MS-EMAT. The MS-EMAT receiver registers only 8.8% of the PPM EMAT receiver voltage. Therefore, the magnetostrictive-induced voltage is less than 8.8% of the total received PPM EMAT signal. This observation indicates that magnetostriction is not significant in the PPM EMAT receiver.

### 3.2. Magnetostriction Model Optimization

The MS-to-PPM and PPM-to-PPM results can be used to calibrate the magnetostriction model with respect to the Lorentz mechanism. The magnetostriction model and normalized experimental data are combined into an optimization problem, with the four empirical factors ($f_{Hot},f_{\alpha },f_{\gamma },f_{\epsilon }$) as variables

(18) $$g_{1}\left(f_{Hot},f_{\alpha },f_{\gamma },f_{\epsilon }\right)=\overset{m}{\underset{i=1}{\sum }}\left|\frac{d_{6y}^{MS}\left(H_{t,i},H_{ot,i},\alpha_{i},\theta_{i},f_{Hot},f_{\alpha },f_{\gamma },f_{\epsilon }\right)}{\left|d_{6y,norm}^{MS}\right|}-\frac{V_{\exp,i}^{MS}/\left|V_{\exp,\mathrm{norm}}^{MS}\right|}{H_{y,i}^{MS}/\left|H_{y,norm}^{MS}\right|}\right|$$

(19) $$g_{2}\left(f_{\gamma },f_{\epsilon }\right)=\left[\overset{n}{\underset{i=1}{\sum }}{\left(-1\right)}^{i+1}\left|\frac{V_{LOR+MS,i}^{PPM}}{V_{exp,i}^{PPM}}\right|\right]-\left|\frac{V_{MS,norm}^{MS}}{V_{exp,norm}^{MS}}\right|$$

where i is the index of a particular experiment run, m is the total number of MS-to-PPM experiments (18), n is the total number of PPM-to-PPM experiments (3), $H_{t,i}$ is the indexed total magnetic field, $H_{ot,i}$ is the indexed static magnetic field, $\alpha_{i}$ is the indexed α angle, $H_{y,i}^{MS}$ is the indexed simulated maximum dynamic y-component field within the plate in the MS-EMAT transmitter, $V_{MS,i}^{MS}$ is the indexed simulated MS-to-PPM maximum absolute receiver voltage, $V_{\exp,i}^{MS}$ is the indexed experimental MS-to-PPM maximum absolute receiver voltage, $V_{LOR+MS,i}^{PPM}$ is the indexed simulated Lorentz and magnetostriction transmitter PPM-to-PPM maximum absolute receiver voltage, $V_{\exp,i}^{MS}$ is the indexed experimental MS-to-PPM maximum absolute receiver voltage, and $V_{\exp,i}^{PPM}$ is the indexed experimental PPM-to-PPM maximum absolute receiver voltage. $d_{6y,norm}$, $V_{exp,norm}^{MS}$, $V_{MS,norm}^{MS}$, and $H_{y,norm}^{MS}$ are the values of $d_{6x,i}$, $V_{\exp,i}^{MS}$, $V_{MS,i}^{MS}$, and $H_{y,i}^{MS}$ at the normalization run (35.1 A input current and 8.92 mm magnet liftoff). $V_{LOR+MS,norm}^{PPM}$ and $V_{\exp,\mathrm{norm}}^{PPM}$ are the values of $V_{LOR+MS,i}^{PPM}$ and $V_{\exp,i}^{PPM}$, respectively, at the highest input current of 20.5 A. $d_{6y}^{MS}$ is Equation (13) with the MS-EMAT formulation.

**Figure 12:**
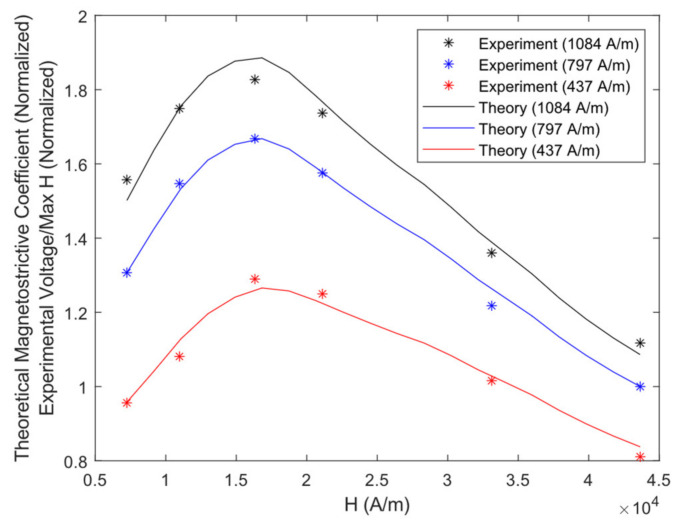
Optimized $d_{6y}^{MS}$ Coefficient Values vs. Maximum Simulated Dynamic Field within the Steel Plate (Theory curve). Experimental Voltage divided by Maximum Simulated Dynamic Field vs. Maximum Simulated Dynamic Field (Experiment points).

Function $g_{1}$ of Equation (18) is minimized using MATLAB’s *fmincon* function to match the calculated $d_{6y}^{MS}$ coefficient with the experimental voltage divided by the dynamic field as closely as possible. $f_{Hot}$ and $f_{\alpha }$ are found from this step, along with an estimate of $f_{\gamma }$ and $f_{\epsilon }$, and the results are shown in Figure 12. Next, function $g_{2}$ of Equation (19) is minimized with all PPM-to-PPM and one MS-to-PPM simulation as well as experimental data to find the final values of $f_{\gamma }$ and $f_{\epsilon }$. The final magnetostriction model factors are tabulated in Table 1.

### 3.3. MS-to-PPM Simulation and Experimental Data

**Figure 13:**
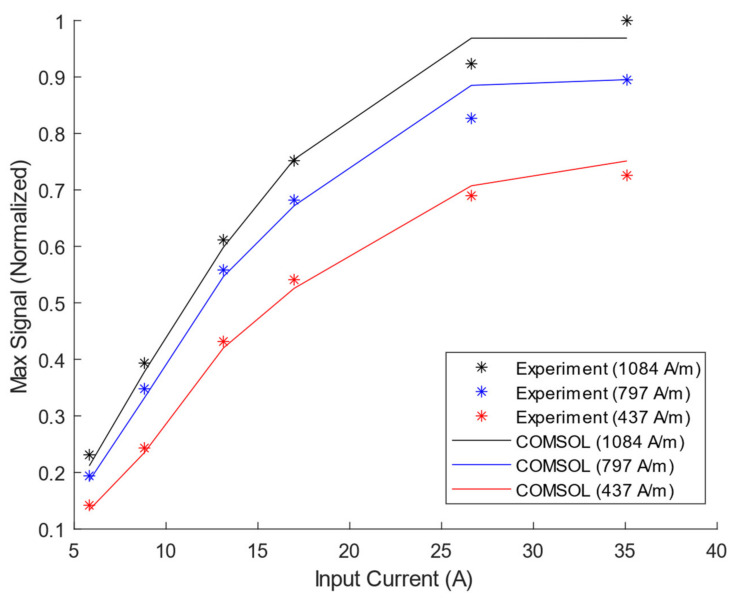
Simulated and Experimental MS-to-PPM Maximum Receiver Voltage on Steel Plate vs. Input Current for three values of Static Field.

The MS-to-PPM experimental and simulated data are summarized in Figure 13, where the maximum time domain receiver voltage amplitude is plotted against the input current. The maximum relative error of all MS-to-PPM simulation results against experiments is 6.6%. The simulated and experiment frequency spectra have a maximum cross-correlation of 0.9973 to 0.9989 for the entire MS-to-PPM set. Therefore, the FEM model can be considered to accurately predict the magnetostriction within the PPM EMAT’s range of operating conditions. Therefore, the model can estimate the relative strength of magnetostriction and Lorentz mechanisms in the PPM transmitter EMAT in the next section.

### 3.4. Magnetostriction within the PPM EMAT Transmitter (PPM-to-PPM EMAT)

**Figure 14:**
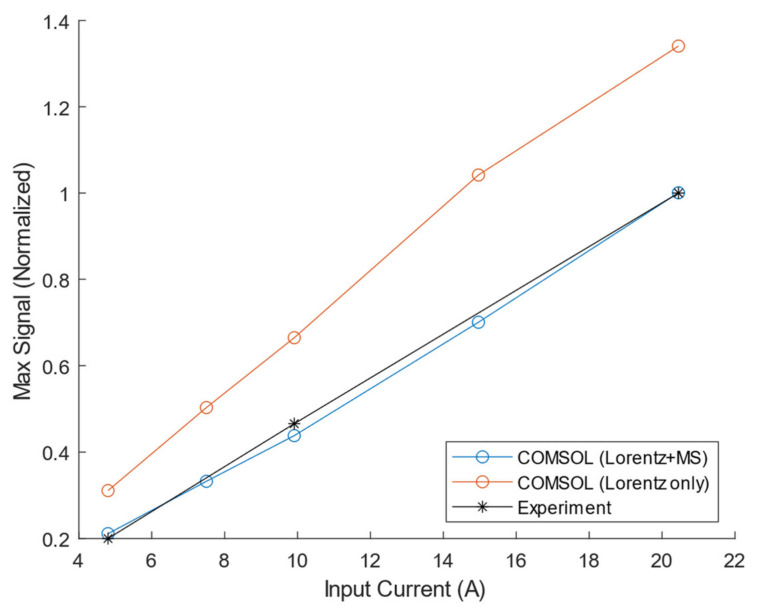
Simulated (either Lorentz only or combined Lorentz and magnetostriction) and Experimental PPM-to-PPM Maximum Receiver Voltage on Steel Plate vs. Input Current.

Figure 14 shows the maximum receiver voltage vs. input current for the COMSOL and experimental results for the PPM-to-PPM system. The total COMSOL simulation containing both Lorentz and magnetostrictive contributions has a maximum deviation of 6.3% from the experimental result. The maximum cross-correlation values between the experimental and simulated absolute frequency spectra range from 0.9965 to 0.9972.

Although separating Lorentz from magnetostriction mechanisms within the PPM EMAT transmitter is difficult in an experiment, it is straightforward in the FE model. By removing the magnetostriction contributions, the received signal for the simulated case of a Lorentz-only PPM transmitter on steel can be estimated. It is observed in FE simulations that the magnetostriction mechanism in the PPM EMAT transmitter *reduces* the wave amplitude by 25.4–34.1%, with an average reduction of 29.2%; this is due to the large phase difference between the two wave generation mechanisms. The PPM EMAT transmitter’s input characteristic is observed to remain linear with the addition of magnetostriction; this is attributed to the PPM EMAT operating mainly within the more linear region of the magnetostriction curve.

**Figure 15:**
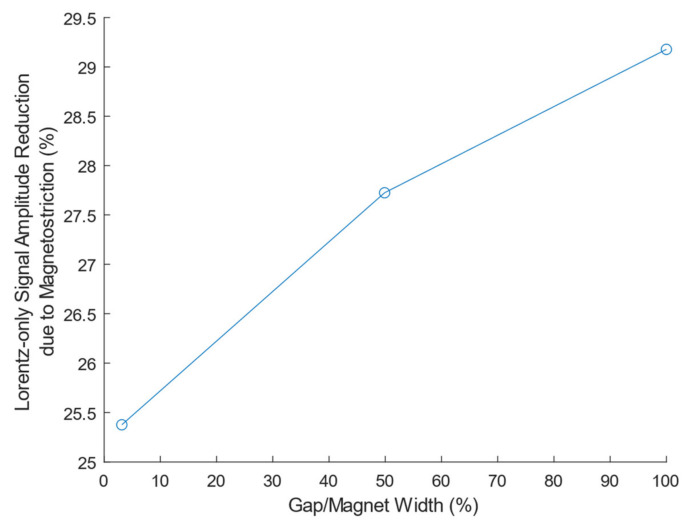
Reduction in the theoretical Lorentz-only PPM-to-PPM Maximum Receiver Voltage due to Magnetostriction vs. PPM EMAT Transmitter Gap/Magnet Width Ratio.

The effect of the relative gap to magnet width in a PPM EMAT transmitter is investigated by simulation. The wavelength, input current, and PPM EMAT receiver geometry remain unchanged, but the PPM EMAT transmitter gap increases by decreasing magnet width. The experimental PPM EMAT is designed with off-the-shelf magnets and has a minimal gap of 3.1% gap/magnet width ratio, which is close to an ideal zero-gap PPM EMAT without ordering custom magnets. The gap is increased for several values (Figure 15), and it is observed that increasing the ratio also increases the magnetostrictive effect on the PPM EMAT transmitter. For example, if the magnet gap is 3% of the magnet width the Lorentz-only signal is reduced by 25.4%. If the gap is increased to 100% of the magnet width (gap equals magnet width), the Lorentz-only signal is instead reduced by 29.2% due to magnetostriction.

## 4. Discussion

The PPM EMAT transmitter generates SH waves with the Lorentz mechanism, but magnetostriction significantly interferes with acoustic wave generation, reducing total output by an average of 29%. There is a negligible magnetostrictive contribution at the PPM EMAT receiver. The 29% figure is lower than the 55% predicted by [13] for the case of a stationary inspection system, but still significant enough to be the source of the +/−20% signal variation when the EMAT system is moved in a direction parallel to that of wave propagation [13]. Thus, magnetostriction affects the PPM EMAT transmitter output in both the stationary and moving case; this increases signal variation in defect-free steel plates.

As magnetostriction cannot be removed from the PPM EMAT without affecting Lorentz, the only SH wave EMAT transmitter that uses a single mechanism is the MS-EMAT. A tradeoff for minimizing the number of active mechanisms is a reduction in signal amplitude. There is a 23% loss in the signal amplitude between the MS-EMAT at 8.92 mm magnet liftoff 35.1 A input current case vs. the PPM EMAT 20.5 A input current. Both are driven by the same pulser and have the same liftoff and static field magnitude. In addition, it is advisable to minimize the gap between each magnet for a given target wavelength.

Provided that a signal produced by an EMAT NDT system is significantly above the noise floor, the focus should be on the variance of the signal due to movement and uneven operating surfaces. Therefore, the ideal EMAT transmitter for reliable nondestructive tests should be minimally sensitive to variations in the electromagnetic fields present or surface finish so that defects have the highest probability of causing any significant signal change. With the information gathered to accomplish the primary objective, the performance of the MS-EMAT can be compared to the theoretical Lorentz-only PPM EMAT in the transmitter case on 1018 steel.

Given a plate with constant material properties, variations in the surface can change the effective liftoff of the EMAT system, which can be approximately modeled as a change in both the static and dynamic fields. A comparison can be made with an MS-EMAT operating at 4.45 mm magnet liftoff and 35.1A input current. The Lorentz-only PPM EMAT shares the same static and dynamic field strengths in the appropriate orientation. Looking at the experimental results of the MS-EMAT transmitter (Figure 14), B-H curve [19], and Lorentz force equation (Equation (1)), the following statements can be made:

- With a 27% reduction in the static field strength, the MS-EMAT’s signal amplitude decreases by 11%, while a Lorentz-only PPM EMAT’s signal amplitude decreases by 18%.
- With a 24% reduction in the dynamic field strength, the MS-EMAT’s signal amplitude decreases by 7.6%, while a Lorentz-only PPM EMAT’s signal amplitude decreases by 24%.
- With a 27% reduction in the static field strength and 24% reduction in the dynamic field strength, the MS-EMAT’s signal amplitude decreases by 17%, while a Lorentz-only PPM EMAT’s signal amplitude decreases by 38% without movement. If the known magnetostrictive effect is now added, the PPM-to-PPM assembly moved from its original position along the wavelength direction will experience a signal reduction of 58%, while the MS-EMAT’s signal loss remains at 17%.

Despite the corresponding MS-EMAT producing lower amplitudes than this study’s PPM EMAT transmitter, the PPM EMAT experiences greater sensitivity to both dynamic field and static field changes, even without magnetostriction (23% vs. 38% in this example, or 58% with the moving magnetostriction effect). It is therefore a favorable tradeoff to use an MS-EMAT rather than PPM EMAT transmitter in industrial settings, where the EMAT may be subjected to forced operating point changes due to uneven surfaces changing the effective liftoff or causing slight movements along the wave propagation direction.

## 5. Conclusions

The PPM EMAT transmitter on steel mainly relies on the Lorentz force to generate SH waves but also induces significant magnetostriction. The magnetostriction effect reduces the total output in the stationary case and is shown in [13] to cause variations in the transmitter–receiver movement case. The competing mechanisms are separated with a semi-empirical numerical model, which incorporates PPM EMAT and MS-EMAT transmitter data at various operating points. Gaps between each magnet in a PPM array must be minimized as increased magnetostriction interference occurs when the relative gap to magnet ratio is increased.

The MS-EMAT transmitter data collected for use in the model has been found to exhibit favorable characteristics at high input currents and low static fields. Changes in either static or dynamic fields have a lesser effect on magnetostriction than the Lorentz mechanism, at the cost of overall signal amplitude. Although this study is not a comprehensive comparison, the MS-EMAT transmitter has shown promise in minimizing signal variation with an acceptable signal amplitude tradeoff. An optimized MS-to-PPM system has the potential to be more reliable in NDT compared to a PPM-to-PPM system.

## Figures and Tables

**Table 1:** Magnetostriction Model Factors.

$f_{Hot}$	$f_{\alpha }$	$f_{\gamma }$	$f_{\epsilon }$
$-4.3102\times 10^{-4}\;m/A$	0.98501	10.061	0.31732
